# Medical thoracoscopy treatment for pleural infections: a systematic review and meta-analysis

**DOI:** 10.1186/s12890-021-01492-9

**Published:** 2021-04-20

**Authors:** Michele Mondoni, Laura Saderi, Federica Trogu, Silvia Terraneo, Paolo Carlucci, Filippo Ghelma, Stefano Centanni, Giovanni Sotgiu

**Affiliations:** 1 Respiratory Unit, ASST Santi Paolo e Carlo, Department of Health Sciences, Università Degli Studi Di Milano, Via Di Rudinì n. 8, 20142 Milan, Italy; 2 Clinical Epidemiology and Medical Statistics Unit, Department of Clinical and Experimental Medicine, University of Sassari, Sassari, Italy; 3 Disabled Advanced Medical Assistance Unit, Department of Health Sciences, San Paolo Hospital, Università Degli Studi Di Milano, Milan, Italy

**Keywords:** Medical thoracoscopy, Pleural infections, Empyema, Pleuroscopy, Parapneumonic effusion, Intra-pleural fibrinolysis

## Abstract

**Background:**

Complicated parapneumonic effusions and empyema represent advanced stages of pleural infections and are characterized by a high mortality. Medical thoracoscopy is a safe and minimally invasive endoscopic technique prescribed to treat severe pleural infections. However, only a few studies evaluated its success rate. A systematic review of observational studies was performed to assess the efficacy of medical thoracoscopy in patients with complicated parapneumonic effusions and empyema, as well as its predictive factors.

**Methods:**

A search of the scientific evidence was carried out using PubMed, EMBASE, and Cochrane Central Register of Controlled Trials. Articles describing observational studies on medical thoracoscopy in patients with parapneumonic effusions and empyema were selected.

**Results:**

Eight studies met the inclusion criteria. The pooled treatment success rate of thoracoscopy was 85% (95% CI 80.0–90.0%; I^2^: 61.8%) when used as first-line intervention or after failure of chest tube. The pooled complication rate was 9.0% (95% CI 6.0–14.0%; I^2^: 58.8%). A pooled difference of treatment success of 9.0% (95% CI 1.0–18.0%) was found when post-thoracoscopy intra-pleural fibrinolysis was prescribed. Pooled success rate was higher in cases with pleural fluid culture negativity (pooled difference: 14.0%; 95% CI 4.0–24.0%).

**Conclusions:**

Medical thoracoscopy is effective and safe when prescribed for complicated parapneumonic effusions and empyema. Bacteriological negativity of pleural effusion specimens and administration of adjuvant intra-pleural fibrinolysis after the procedure are associated with a higher success rate.

**Supplementary Information:**

The online version contains supplementary material available at 10.1186/s12890-021-01492-9.

## Background

Pleural infections are highly incident medical conditions, which frequently complicate both community- and healthcare-associated pneumonia [1–3].

Complicated parapneumonic effusion (CPE) (i.e., pleural infection with at least one of the following criteria: pH < 7.2, LDH > 1000 IU/l or glucose < 60 mg/dL) and empyema (i.e., pus in the pleural space or positive gram stain/culture for pathogenic organisms) represent advanced stages of disease, characterized by high mortality [2–5].

International guidelines suggest antibiotic therapy and early drainage of the pleural space by chest tube; surgery may be recommended when patients fail medical therapy and/or in case of organized empyema with extensive pleural fibrosis requiring decortication [4, 6].

Medical thoracoscopy (i.e., thoracoscopy performed with local anaesthesia under conscious sedation) is a safe and minimally invasive endoscopic technique, which is prescribed for several pleural diseases (e.g. diagnosis of pleural effusions of unknown aetiology or suspected for malignancy, talc poudrage pleurodesis in pleural neoplasms and pneumothorax) [7–9]. It shows several advantages if compared with conventional medical therapy, particularly in cases of multi-loculated pleural effusion [7–9]. Indeed, it can mechanically disrupt fibrin adhesions, can help position chest tubes under direct vision and perform pleural biopsy [7–9]. Current guidelines do not recommend its use for the above-mentioned indications, following the absence of any evidence from randomized controlled trials on the efficacy in the management of pleural infections [4, 7].

A limited number of studies specifically assessed the therapeutic success of medical thoracoscopy in cases of CPE and empyema [10–18].

The aim of the present systematic review was to describe the findings of the available scientific evidence in order to provide a pooled estimate of therapeutic efficacy of medical thoracoscopy in CPE and empyema, and to identify the main predictive factors of a successful procedure.

## Methods

### Search strategy

Observational studies on the therapeutic use of medical thoracoscopy in patients with CPE and empyema were searched in the search engines PubMed, Cochrane Central Register of Controlled Trials, and EMBASE, from their inception to 30th June 2020.

To retrieve the scientific evidence from the above-mentioned databases strings were created using the following single and combined key-words: “thoracoscopy”, “medical thoracoscopy”, “empyema”, “complicated parapneumonic effusion”, “loculated parapneumonic effusion”, “CPE”, “pleural infection”, and “pleural effusion”.

Reviews on this topic or similar topics, as well as their list of references, were carefully evaluated to identify pertinent manuscripts.

Conference proceedings and abstracts of national and international congresses were basically excluded being unreliable for the poor information provided in the methods and in the results sections.

The systematic review was registered at PROSPERO with the number CRD42018096193.

### Study selection

The selected epidemiological studies were aimed at evaluating the therapeutic efficacy of medical thoracoscopy in patients with CPE or empyema, as well as the risk factors associated with a successful intervention.

Articles were excluded based on one of the following criteria: (1) unclear primary and secondary outcomes; (2) thoracoscopy adopted for diagnostic purposes; (3) studies performed in animals; (4) case-reports or case-series which enrolled less than 10 patients; (5) editorials, correspondences, reviews; (6) languages other than English; (7) observational studies evaluating thoracoscopy for the treatment of complicated parapneumonic effusions and empyema performed with general anesthesia and/or endotracheal intubation and/or involving other type of anesthesia different from local anesthesia; 8) experimental studies.

Two Authors (L.S. and F.T.) carefully and independently evaluated titles and abstracts of the records for the selection of the full texts, which were assessed for their eligibility by the same Authors.

Discrepancies during the selection of the articles and extraction of the variables were solved by the intervention of a third Author (G.S.).

### Data extraction

Qualitative and quantitative variables were collected in an ad hoc electronic form.

The following variables were collected: first author of the article; title of the article; year when the study was published; year/s when the study was conducted; epidemiological study design; country/ies where the study was carried out; sample size; age; gender; therapeutic thoracoscopy for empyema; microbiological diagnosis; diagnosis of tuberculosis (TB); etiology of pleural effusion; treatment success; tube duration; assessment of successful intervention; complications of medical thoracoscopy; mortality; type of radiological screening; radiological findings; pleural infection stage; type of intra-pleural fibrinolytic therapy (IPFT); duration of symptoms; entry points; type of thoracoscope; tool adopted for fibrinous septae disruption; chest tube; post-procedure negative pressure suction.

Data independently extracted by the above-mentioned Authors were cross-checked for the detection of inconsistencies. The inter-rater agreement was ~ 100% because no significant discrepancies were found.

No ethical approval was requested to the ethical committee because anonymized and aggregated data were retrieved from the selected articles.

### Study quality assessment

Inter-rated agreement was ~ 100% for the phases of study selection and data extraction and the few inconsistencies were solved by consensus and with the support of a third senior Author (G.S.).

Guidelines of the Preferred Reporting Items for Systematic Reviews and Meta-Analysis (PRISMA) were followed to guide the process of the systematic review and meta-analysis.

The scientific quality of the observational studies was evaluated according to the Scottish Intercollegiate Guidelines Network.

### Statistical analysis

A descriptive analysis of the qualitative and quantitative variables was carried out: absolute and relative (percentage) frequencies and means (range) were used to summarize qualitative and quantitative variables, respectively.

Forest plots were used to describe between-study variability of point and interval (95% confidence interval, CI) estimates, as well as the weight of the sample size of recruited studies. Pooled and heterogeneity indicators were adopted to summarize key study variables. The I^2^ indicator (low, medium, and high heterogeneity expressed as < 25%, ≥ 25–< 50%, ≥ 50%, respectively) showed the association between true variability and overall variation.

Fixed and random-effects models were chosen depending on the between-study heterogeneity.

Publication bias was graphically evaluated with bias assessment plots, as well as with the Egger weighted regression test. A two-tailed p value less than 0.05 was deemed statistically significant. The statistical softwares were Stata version 16 (StatsCorp, Texas, USA) and StatsDirect version 3.1.12 (StatsDirect Ltd).

## Results

### Study selection

**Fig. 1:**
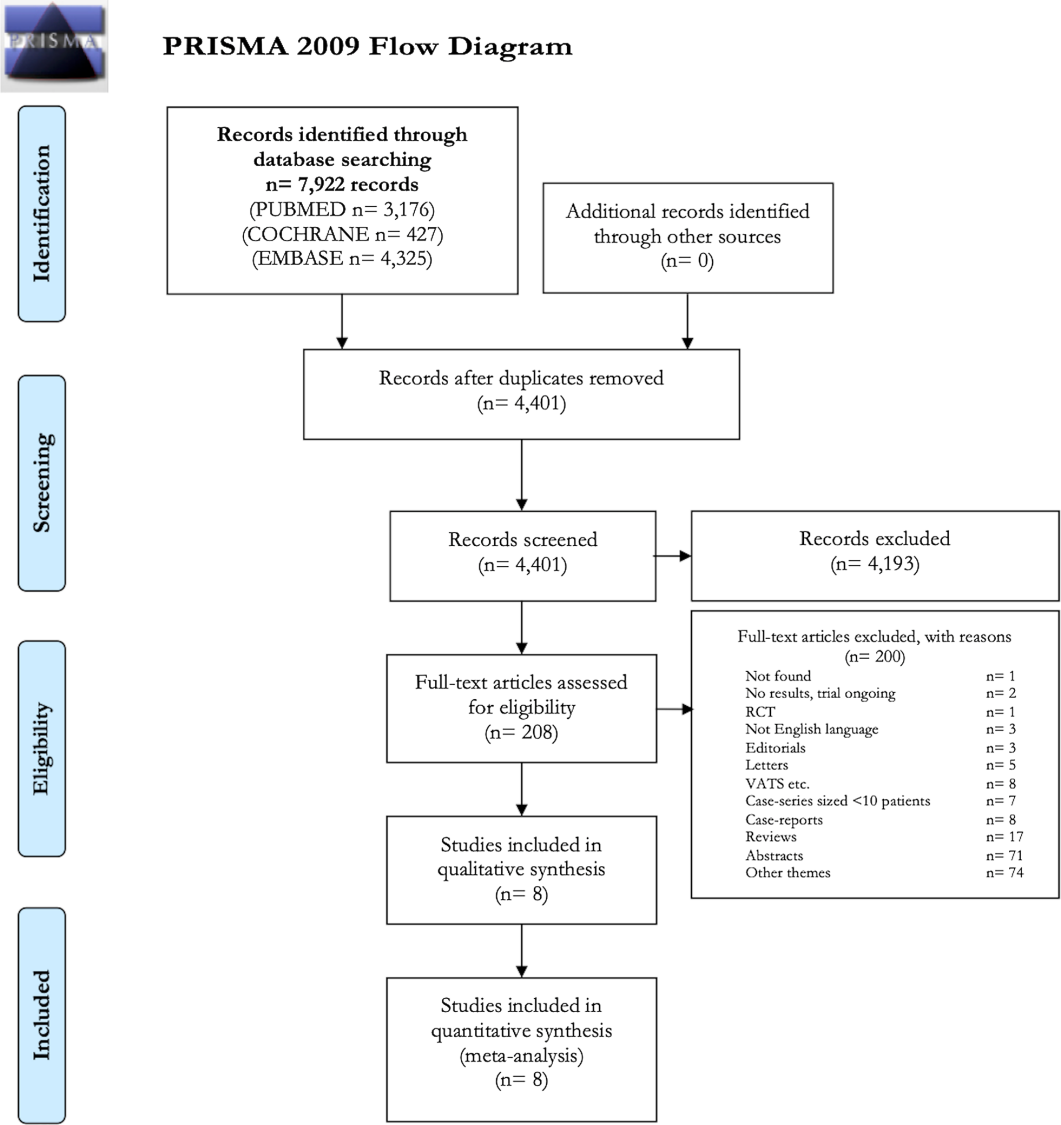
PRISMA (Preferred Reporting Items for Systematic Reviews and Meta-Analyses) 2009 flow diagram

The search of the electronic databases found 7,922 records (Fig. 1). A total of eight studies were selected for the qualitative and quantitative analysis; 200 studies were excluded for the following reasons: other topics (n = 74), only abstract available (n = 71), reviews (n = 17), case-reports (n = 8), case-series with less than ten patients (n = 7), Video Assisted Thoracoscopic Surgery (VATS) (n = 8), letters (n = 5), editorials (n = 3), articles written in languages than English (n = 3), randomized controlled trials (n = 1), studies without any results (n = 2), and full-text not available (n = 1).

### Characteristics of the selected studies

The articles describe studies carried out between 1989 [11] and 2016 [15, 17]. They were published between 1997 [10] and 2018 [17] (Table 1).

**Table 1:** Summary of the included studies

Author	Title	Study year	Type of study	Mono/multicentre	Setting	Study period	Sample size, n	Therapeutic thoracoscopy, n (%)
Solèr [10]	Treatment of early parapneumonic empyema by "medical" thoracoscopy	1997	Case series	Monocentre	Switzerland	Dec 1992–Sep 1994	16	16 (100.0)
Brutsche [11]	Treatment of sonographically stratified multiloculated thoracic empyema by medical thoracoscopy	2005	Retrospective study	Multicentre	Italy/Switzerland	1989–2003	127	127 (100.0)
Ravaglia [12]	Is medical thoracoscopy efficient in the management of multiloculated and organized thoracic empyema?	2012	Retrospective observational study	Monocentre	Italy	Jul 2005–Feb 2011	41	41 (100.0)
Ohuchi [13]	Single trocar thoracoscopy under local anesthesia for pleural space infection	2014	Case series	Monocentre	Japan	Jan 2000–Dec 2012	29	29 (100.0)
Xiong [14]	Role of medical thoracoscopy in the treatment of tuberculous pleural effusion	2016	Retrospective study	Monocentre	China	Jan 2009–Jun 2014	430	65 (15.1)
Abo-El-maged [15]	Safety and efficacy of medical thoracoscopy in the management of loculated thoracic empyema	2017	Retrospective observational study	Monocentre	Egypt	Oct 2015–Aug 2016	30	30 (100.0)
Hardavella [16]	Hippocrates quoted “If an Empyema Does Not Rupture, Death Will Occur”. Is medical thoracoscopy able to make it rupture safely?	2017	Retrospective observational study	Multicentre	UK/Greece	Jan 2001–Nov 2014	84	84 (100.0)
Sumalani [17]	Role of medical thoracoscopy in the management of multiloculated empyema	2018	Case series	Monocentre	Pakistan	Sep 2014–Aug 2016	160	160 (100.0)

*US* United States, *UK* United Kingdom

The following countries were involved in the selected studies: Switzerland (2, 22.2%) [10, 11], Italy (2, 22.2%)[11, 12], Japan (1, 11.1%) [13], China (1, 11.1%) [14], Egypt (1, 11.1%) [15], UK (1, 11.1%) [16], Greece (1, 11.1%)[16], and Pakistan (1, 11.1%)[17]. Studies were mono-center in most of the cases (6, 66.7%) [10, 12–15, 17], whereas multi-center in only two (25%)[10, 16] studies. Three (33.3%) [10, 13, 17] case-series recruiting ≥ 10 patients were selected; five (62.5%) [11, 12, 14–16] studies were observational and retrospective, without providing further epidemiological details.

### Characteristics of the study samples

Sample size of the selected studies ranged from 16 [10] to 430 [14] patients (Table 1). The majority of the studies (7, 77.8%) [10, 12, 13, 15–17] described the use of thoracoscopy for the management of empyema, whereas one article described this scope of thoracoscopy only in 15.1% [14] of their patients.

The mean (range) age of the recruited patients in a single study was 51.7 (6–93[9, 10, 12]) years. The proportion of males in every single research ranged from 62.5 [9] to 82.9% [12].

Microbiological diagnosis was carried out in 100% of the patients in only two (22.2%) [14, 17] studies; however, in two (25%) studies the proportion was less than 50% (i.e., 45.7% [11], and 40.0% [15]) (Additional file 1: Table S1). TB diagnosis was performed in only four (44.4%) [11, 12, 14, 17] studies. Infections caused by Gram-positive and -negative bacteria were found in 5 (55.6%) [10–13, 15] and 5 (55.6%) [10–13, 15] studies, respectively. The proportion of infections caused by Gram-positive bacteria in every study ranged from 9.8 [12] to 41.4% [13], whereas the proportion of infections caused by Gram-negatives varied from 7.9 [11] to 14.6% [12].

A diagnosis of malignancy in empyema specimens was performed in four (44.4%) [11, 13, 15, 17] studies: the percentage of cases ranged from 0.3 [15] to 12.5% [17].

**Fig. 2:**
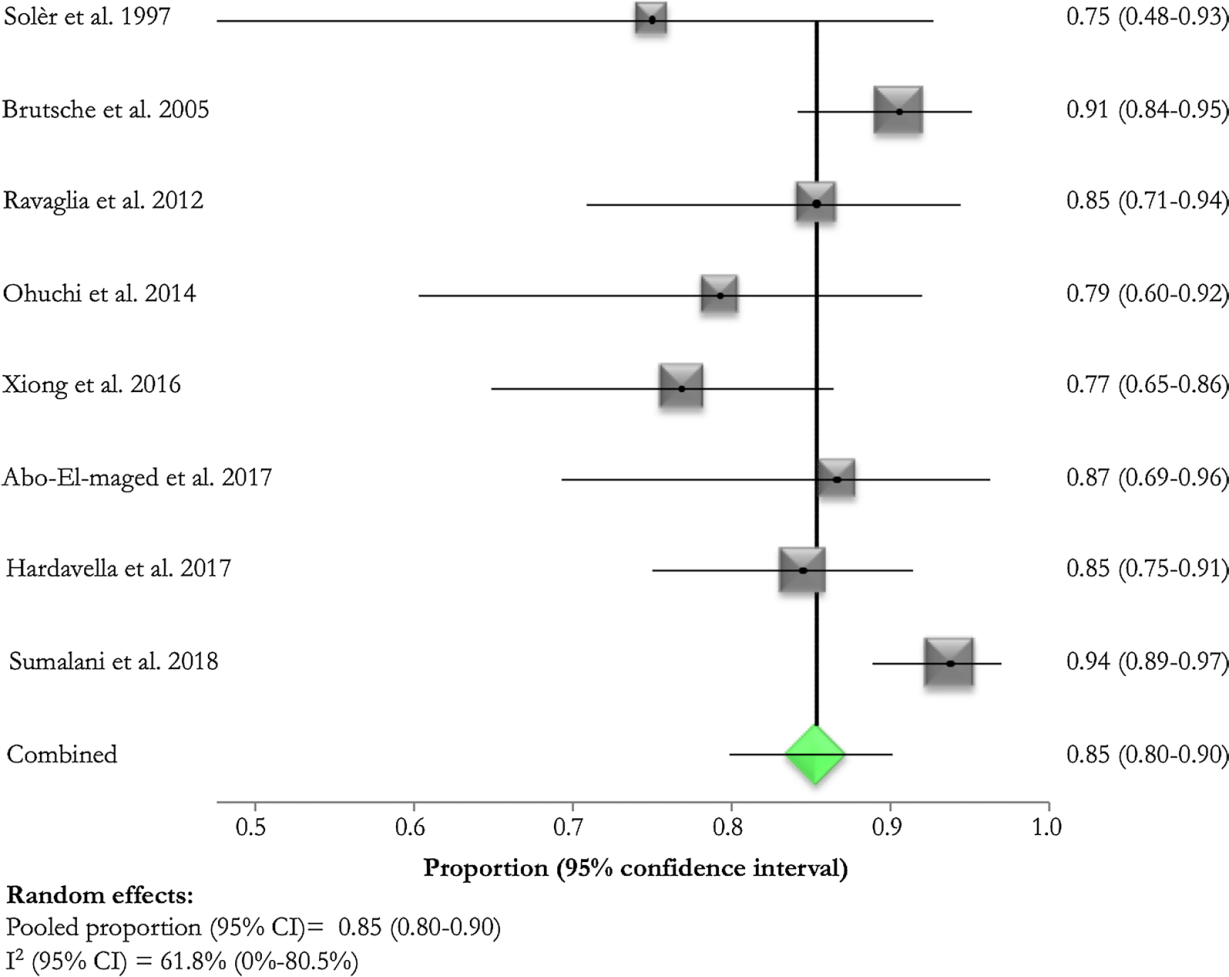
Treatment success rate of medical thoracoscopy in complicated parapneumonic effusion and empyema

Treatment success rate was described in all selected studies [10–18]: it varied from 75.0 [10] to 94% [17] (Table 2). Pooled treatment success rate was 85% (95% CI 80.0–90.0%; I^2^: 61.8%) (Fig. 2). Pooled treatment success rate in Gram-positive and -negative infections was 74.0% (95% CI 61–86%; I^2^: 16.1%) and 77.0% (95% CI 54.0–93.0%; I^2^: 0.0%) respectively. Definition of success depended on the absence of further surgical interventions in four (50%) [9, 10, 14, 16] studies. Other three (33.3%) [12, 13, 17] studies defined a successful thoracoscopy based on a radiological resolution and objective evidence of sepsis resolution; one (11.1%) [15] study defined the technique successful following clinical and radiological improvement (Table 2).

**Table 2:** Main outcomes of the included studies

Study	Treatment success	Assessment of successful intervention
Solèr et al. [10]	12 (75)	No further surgical intervention
Brutsche et al. [11]	115 (90.6)	No further intervention
Ravaglia et al. [12]	35 (85.4)	Radiological resolution, objective evidence of sepsis resolution
Ohuchi et al. [13]	23 (79.3)	Radiological resolution, objective evidence of sepsis resolution
Xiong et al. [14]	50 (76.9)	No further surgical intervention
Abo-El-maged et al. [15]	26 (86.7)	Clinical and radiological improvement
Hardavella et al. [16]	71 (84.5)	No further surgical intervention
Sumalani et al. [17]	150 (93.8)	Radiological resolution

Complications were described by seven (77.8%) [11–17] studies: the rate of complications ranged from 3.1 [14] to 26.7% [15] (Table 3). Pooled complication rate was 9.0% (95% CI 6.0–14.0%; I^2^: 58.8%). The most frequent adverse event was air leakage: it was reported by three (33.3%) studies [11, 16, 17].

**Table 3:** Complications following medical thoracoscopy

Study	n (%)	Complications detail	n (%)
Solèr et al. [10]	–	–	–
Brutsche et al. [11]	12/127 (9.5)	Air leak of 3–7 days	9/127 (7.1)
		Subcutaneous emphysema	3/127 (2.4)
Ravaglia et al. [12]	3/41 (7.3)	Cutaneous fistula	1/41 (2.4)
		Residual pneumothorax	1/41 (2.4)
		Haemothorax	1/41 (2.4)
Ohuchi et al. [13]	1/29 (3.5)	Port insertion to the abdominal cavity	1/29 (3.5)
Xiong et al. [14]	2/65 (3.1)	Subcutaneous tuberculous abscess	2/65 (3.1)
Abo-El-maged et al. [15]	8/30 (26.7)	Failure of lung expand	3 (10.0)
		Broncho-pleural fistula	1 (3.3)
		Surgical emphysema	4 (13.3)
Hardavella et al. [16]	11/84 (13.1)	Atrial fibrillation	4/84 (4.8)
		Port site infection	3/84 (3.6)
		Air leak of 3–7 days	2/84 (2.4)
		Postoperative pneumoniae	1/84 (1.2)
		Deep vein thrombosis	1/84 (1.2)
Sumalani et al. [17]	10/160 (6.3)	Persistent air leak	9/160 (5.6)
		Death	1/160 (0.6)

Chest x-ray and CT scan were adopted to screen the intervention in 1 (11.1%) [13] study, whereas one (11.1%) [12] study used CT scan and chest ultrasounds (Additional file 1: Table S2). Two (22.2%) [10, 11] studies reported the use of only ultrasonography, whereas four (44.4%) [14–17] studies used all radiological tools previously mentioned. The most prevalent radiological findings were multi-loculated (7, 77.8%, studies [11–17]), free flowing effusion (3, 33.3%, studies [12, 15, 16]), and organized effusion (2, 22.2%, studies [12, 14]). Staging of pleural infections was performed in all studies [10, 17]: empyema was described in 8 (88.9%) studies [10–13, 15–17], whereas complicated parapneumonic effusion in 3 (33.3%) studies [10, 13, 14].

Entry-points were described in three (37.5%) [14, 16, 17] studies: they ranged from one [14, 16, 17] to two [17]. Thoracoscopy was used as first-line intervention and after failure of chest tube in four (44.4%) [13–16] and in four (44.4%) [10–12, 17] studies, respectively (Table 4). The type of thoracoscope was semi-rigid [13] or rigid [9–12, 14–16]; only one (12.5%)[17] study did not report on the characteristics of the thoracoscope. The instrument adopted for fibrinous septae disruption was biopsy forceps in eight (88.9%) studies [10–17]. Chest tube size ranged from 20 [12] to 32 F [12, 17]. Post-procedure negative pressure suction was adopted in 6 (66.7%) studies [10–14, 17]. Mean (range) duration of tube positioning was 8.2 (2–18 [10, 12, 13, 16]) days, although it was reported only by five (55.6%) [10, 12–14, 16] manuscripts.

**Table 4:** Main characteristics of the thoracoscopic procedures

Study	Timing of thoracoscopy	Entry points, *n*	Type of thoracoscope	Instrument used for fibrinous septae disruption	Chest tube (size)	Mean (range) tube duration (days)	Post-procedural negative pressure suction application (cmH20)
Solèr et al. [10]	After failed chest tube attempt	–	Rigid	Biopsy forceps	–	6 (2–14)	Yes (− 25 to 50 cmH20)
Brutsche et al. [11]	After failed chest tube attempt (˜one-third of the total sample size”)	–	Rigid	Biopsy forceps	24 or 28F	–	Yes (− 20 cmH20)
Ravaglia et al. [12]	After failed chest tube attempt in 9/41 subjects	–	Rigid	Biopsy forceps	20–32F	7.9 (2–17)	Yes (− 20 cmH20)
Ohuchi et al. [13]	First-line	–	Semi-rigid	Biopsy forceps	24F double lumen	9.2 (3–18)	Yes (− 15 cmH20)
Xiong et al. [14]	First-line	1	Rigid	Biopsy forceps	–	11 (5–15)	Yes (− 20 cmH20)
Abo-El-maged et al. [15]	First-line (27 patients) and second line in patients who failed chest tube drainage (3 patients)	–	Rigid	Biopsy forceps	26–28F	–	–
Hardavella et al. [16]	First-line	1	Rigid	Biopsy forceps	–	7 (2–17)	–
Sumalani et al. [17]	Prolonged presentation of empyema (> 30 days) No response to antibiotics therapy Failure of complete drainage by tube thoracostomy	1 or 2	–	Biopsy forceps	28–32F	–	Yes (− 20 cmH20)

*F* French, *cmH20* centimeters of water

Three (33.3%) [11–13] studies evaluated the treatment success rate in patients exposed to post-thoracoscopy intra-pleural fibrinolysis: it ranged from 81.8 [13] to 96.8% [11] Brutsche et al. administered 250.000 U of streptokinase or 100,000 U of urokinase diluted in 100 ml of normal saline solution once daily during 3–5 days, in the presence of depots of fibrin after thoracoscopy [11]; Ravaglia et al. administered 100.000 U of urokinase diluted in 100 ml of saline solution once daily for 3–5 days in case of multiloculated and organized empyema [12], while Ohuchi et al. employed urokinase or fibrinolysin/deoxyribonuclease (240.000 U diluted in 40 ml of normal saline solution once daily) in case of residual spaces after thoracoscopy [13] (Additional file 1: Table S3).

**Fig. 3:**
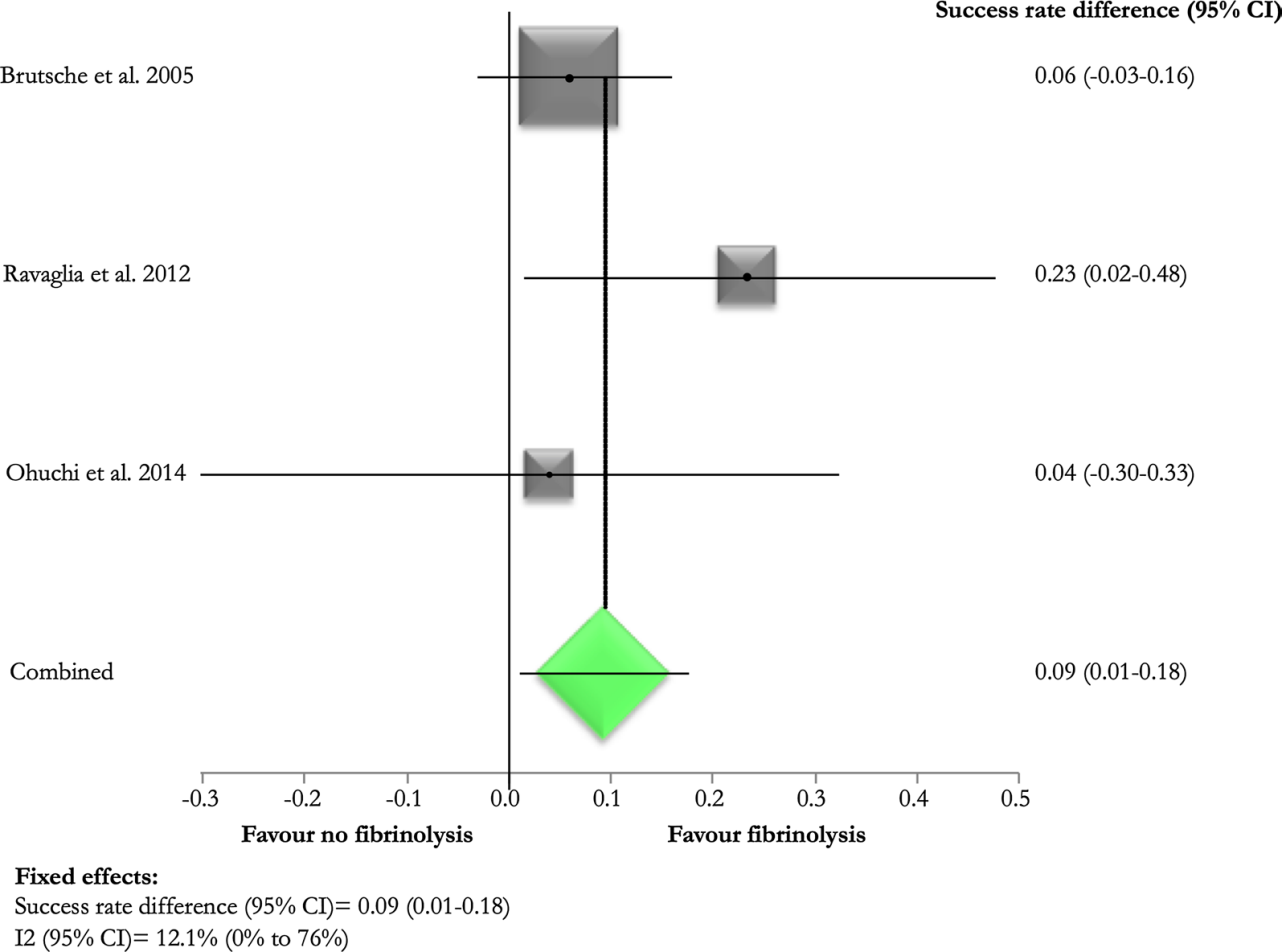
Success rate differences according to the administration of post-thoracoscopy intra-pleural fibrinolysis

**Fig. 4:**
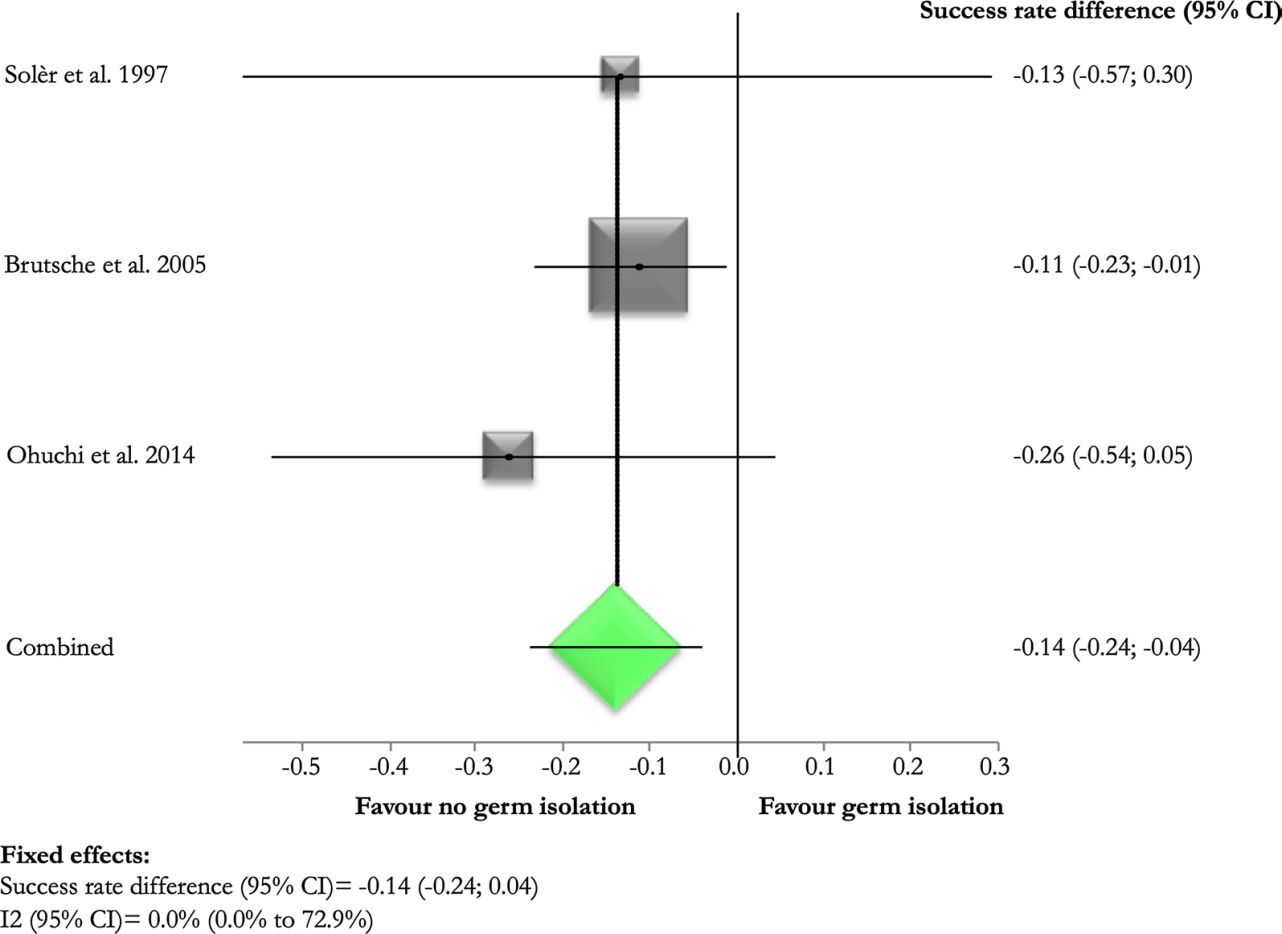
Success rate differences according to detection of bacteriological infection

Pooled difference of treatment success rate between those exposed and not exposed to fibrinolysis was 9% (95% CI 1.0–18.0%) in favour of fibrinolysis (Fig. 3). Pooled success rate was higher in those without any bacteriological detection (pooled difference 14.0%; 95% CI 4.0–24.0%) (Fig. 4).

Quality assessment found a high risk of bias in the majority of the recruited studies (8, 88.9% [10–13, 15–17]) (Additional file 1: Table S4).

## Discussion

The present systematic review and meta-analysis provides for the first time a comprehensive description of the main results published in studies which evaluated the therapeutic efficacy of medical thoracoscopy for CPE and empyema. Overall, the pooled therapeutic success rate (85.0%) was high, and the safety profile of the technique when performed as first-line procedure or after a chest tube treatment failure was good (pooled complication rate of 9.0%). The findings are clinically relevant: the vast majority of the patients with complicated pleural infections who underwent this technique recover and do not need more invasive surgical procedures.

Predictors of high effectiveness included the negative bacteriological detection and the administration of intra-pleural fibrinolysis after the procedure.

Previous studies showed conflicting findings on the prognosis of culture-positive pleural effusions, which could be associated with a higher bacterial load [11, 18–20]. Brutsche et al. demonstrated a higher treatment failure of medical thoracoscopy in culture positive effusions and empyema [11]. Okikor et al. showed a longer duration of pleural drainage and hospital stay and more frequent complications in patients with culture-positive organized empyema who underwent thoracotomy and decortication, without any differences in achieving lung re-expansion [18]. Notably, Khemasuwan et al. did not prove a role of culture positivity on treatment outcomes in 84 patients with CPE/empyema treated with chest tube and intra-pleural fibrinolytic therapy [19].

Our data confirm the findings of Brutsche et al., with a higher success rate of the technique in case of culture negative pleural infections [11]. No difference in therapeutic efficacy was recorded between different germ type infections (i.e. gram positive versus gram negative) [11].

International guidelines do not suggest the routine use of IPFT in the absence of a clear benefit over placebo in terms of reduction of mortality, referral for surgery, length of hospital stay, and radiological improvement [4, 6]. They postulate their possible administration in case of symptomatic, persistent, multi-loculated pleural effusions in patients whose conditions do not allow the immediate implementation of surgical procedures (e.g., comorbidities) [4].

Notably, a more recent randomized clinical trial (i.e. MIST2 trial) and a meta-analysis showed that intrapleural fibrinolytic therapy, administered through chest tube, was associated with a reduced frequency of surgical interventions and treatment failure; however, mortality rate did not significantly change [21, 22].

A recent small randomized clinical trial, which enrolled 32 patients with pleural infections was designed to compare the length of hospital stay between medical thoracoscopy and chest tube with IPFT. It failed to detect a significant difference in therapeutic success between these techniques (75.0% vs. 81.3%, respectively). However, the study was not powered to detect a difference for this outcome [23].

Three studies described the post-thoracoscopy administration of IPFT in patients with multi-loculated pleural effusions and/or in case of persistent fibrin depots following the endoscopic procedure [11–13].

Pooled estimate shows a slightly higher therapeutic success after combining medical thoracoscopy and intra-pleural fibrinolytics administered after the procedure. This might suggest a potential advantage related to IPFT after the mechanical disruption of major pleural adhesions by the thoracoscopic forceps.

Half of the studies included in the present meta-analysis describe the use of thoracoscopy as first-line technique, instead of second-line therapeutic option after chest tube failure. Since this approach does not reflect the current standard of care, mainly in those patients with free-flowing pleural effusion, it may represent a limitation in changing clinical practice.

Four studies described the diagnosis of malignancy associated to empyema. Local anesthetic thoracoscopy may also play an important diagnostic role in difficult-to-treat pleural infections. Indeed, pleural biopsies help collect additional tissue samples for microbiological and histopathological examinations [11, 15, 17].

Some methodological limitations of the present review could be found.

The quality of the study design is poor: the quality assessment found a high risk of bias. The selected studies were observational and confounding factors and selection bias could hinder the reliability of the findings. Some important variables were not collected or described in the selected studies: this can under-or over-estimate some point estimates. Furthermore, standard operating procedures could be different: the available information retrieved from the individual studies cannot be sufficient to discriminate any procedural differences which can affect the findings. The timing of the procedure is not clearly defined: this important variable could explain some results described by the selected studies. Finally, several studies have adopted various definitions of treatment success for medical thoracoscopy.

## Conclusions

In conclusion, this systematic review and meta-analysis shows that medical thoracoscopy is an effective and safe minimally invasive technique for the treatment of complicated parapneumonic effusion and empyema. The bacteriological negativity and the administration of adjuvant IPFT immediately after the procedure are associated with better therapeutic outcomes.

Despite the study limitations, our findings could be the scientific basis of new recommendations: current international guidelines do not suggest medical thoracoscopy in the therapeutic work-up of pleural infections.

Ongoing randomized controlled trials (ClinicalTrials.gov Identifiers: NCT03468933; NCT03859206) will directly compare cost-effectiveness of medical thoracoscopy with standard medical treatment (i.e., chest tube with and without IPFT).

The good performance and the optimal safety profile could change the therapeutic landscape of pleural infections: thoracoscopy could be the first-line technique in multi-loculated CPE and empyema, mainly in frail patients (e.g., elderly with comorbidities) who cannot undergo general anesthesia and surgical procedures [9, 12, 24].

Future studies are needed to assess the optimal timing of medical thoracoscopy in the management of advanced pleural infections.

## Supplementary Information

**Additional file 1.** **Table 1 supplement.** Microbiological diagnosis of pleural effusion. **Table 2 supplement.** Chest imaging findings for patients’ classification. **Table 3 supplement.** Fibrinolysis treatment. **Table 4 supplement.** Checklist for cohort studies (1), according to the Scottish Intercollegiate Guidelines Network.

## Data Availability

The datasets used and/or analyzed during the current study are available from the corresponding author on reasonable request.

## Abbreviations

CPE: Complicated parapneumonic effusion
TB: Tuberculosis
VATS: Video assisted thoracoscopic surgery
IPFT: Intra-pleural fibrinolytic therapy
