# Supplementary material for: Medical thoracoscopy treatment for pleural infections: a systematic review and meta-analysis
Source: BMC Pulm Med. 2021 Apr 20;21:127. doi: 10.1186/s12890-021-01492-9 (PMC8056545; doi:10.1186/s12890-021-01492-9)
Supplement: Supplementary file 1 — Additional file 1. Table 1 supplement. Microbiological diagnosis of pleural effusion. Table 2 supplement. Chest imaging findings for patients’ classification. Table 3 supplement. Fibrinolysis treatment. Table 4 supplement. Checklist for cohort studies (1), according to the Scottish Intercollegiate Guidelines Network. [file 12890_2021_1492_MOESM1_ESM.docx]

**Supplementary material**

**Table 1 supplement. Microbiological diagnosis of pleural effusion.**

| **Study** | **Microbiological diagnosis, n (%)** | **Tuberculosis, n (%)** | **Gram +, n (%)** | **Gram -, n (%)** |
| --- | --- | --- | --- | --- |
| Solèr et al. 1997[10] | 6/16 (37.5) | - | 4 (25.0) | 2 (12.5) |
| Brutsche et al. 2005[11] | 58/127 (45.7) | 1/127 (0.8) | 27 (21.3) | 10 (7.9) |
| Ravaglia et al. 2012[12] | 24/41 (58.5) | 11/41 (26.8) | 4 (9.8) | 6 (14.6) |
| Ohuchi et al. 2014[13] | 15/29 (51.7) | - | 12 (41.4) | 3 (10.3) |
| Xiong et al. 2016[14] | 430/430 (100.0) | 430/430 (100.0) | - | - |
| Abo-El-maged et al. 2017[15] | 12/30 (40.0) | - | 4 (13.3) | 6 (10.0) |
| Hardavella et al. 2017[16] | - | - | - | - |
| Sumalani et al. 2018[17] | 160/160 (100.0) | 102/160 (63.8) | - | - |

**Table 2 supplement. Chest imaging findings for patients’ classification.**

| **Study** | **Chest imaging method for classification** | **Chest imaging findings** | **Pleural infection stage** |
| --- | --- | --- | --- |
| Solèr et al. 1997[10] | Ultrasonography |  | Complicated parapneumonic effusions and empyemas |
| Brutsche et al. 2005[11] | Ultrasonography | Multiloculated (n= 127) | Empyemas |
| Ravaglia et al. 2012[12] | CT scan and ultrasonography | Multiloculated (n= 24)  Organized (n= 8)  Free-flowing effusion (n= 9) | Empyemas |
| Ohuchi et al. 2014[13] | Chest X ray and CT scan | Multiloculated (n= 8)  Complicated parapneumonic effusion (n= 21) | Complicated parapneumonic effusions and empyemas |
| Xiong et al. 2016[14] | Chest X ray, ultrasonography and/or CT scan | Multiloculated (n= 28)  Organized (n= 37) | Complicated parapneumonic effusions |
| Abo-El-maged et al. 2017[15] | Chest X ray, CT, ultrasonography | Multiloculated (n= 9)  Free-flowing (n= 21) | Empyemas |
| Hardavella et al. 2017[16] | Chest X ray, CT scan and ultrasonography | Multiloculated (n= 75)  Free-flowing (n= 9) | Empyemas |
| Sumalani et al. 2018[17] | Chest X ray, CT scan and ultrasonography | Multiloculated (n= 160) | Empyemas |

CT: computed tomography

**Table 3 supplement. Fibrinolysis treatment.**

| **Study** | **Patients treated with fibrinolysis, n (%)** | **Success in patients treated with fibrinolysis, n (%)** | **Success in patients non treated with fibrinolysis, n (%)** | **Type of fibrinolytic** | **Dosage** | **Indication** |
| --- | --- | --- | --- | --- | --- | --- |
| Brutsche et al. 2005[11] | 62/127 (48.8) | 60/62 (96.8) | 59/65 (90.8) | Streptokinase or urokinase | 250,000 U of streptokinase or 100,000 U of  urokinase diluted in 100 ml of normal saline solution once daily (3 to 5 days) | Depots of fibrin after thoracoscopy |
| Ravaglia et al. 2012[12] | 23/41 (56.1) | 22/23 (95.7) | 13/18 (72.2) | Urokinase | 100,000 U diluted in 100 ml of saline solution once daily or 3-5 days | Multiloculated  and organized empyema |
| Ohuchi et al. 2014[13] | 11/21 (52.4) | 9/11 (81.8) | 14/18 (77.8) | Urokinase or fibrinolysin/deoxyribonuclease | 240,000 U  diluted in 40 ml of normal saline solution once daily | In cases of residual spaces after thoracoscopy |

**Table 4 supplement. Checklist for cohort studies ^(1)^, according to the Scottish Intercollegiate Guidelines Network.**

| **Study** | **Q1** | **Q2** | **Q3** | **Q4** | **Q5** | **Score** | **Grade of evidence ^(2)^** |
| --- | --- | --- | --- | --- | --- | --- | --- |
| Solèr et al. 1997[10] | No | Yes | No | No | No | 1 | - |
| Brutsche et al. 2005[11] | Yes | Yes | No | Yes | No | 3 |  |
| Ravaglia et al. 2012[12] | Yes | No | No | No | No | 1 | - |
| Ohuchi et al. 2014[13] | Yes | No | No | No | No | 1 | - |
| Xiong et al. 2016[14] | Yes | Yes | No | Yes | No | 3 |  |
| Abo-El-maged et al. 2017[15] | Yes | No | No | No | No | 1 | - |
| Hardavella et al. 2017[16] | Yes | No | No | No | No | 1 | - |
| Sumalani et al. 2018[17] | Yes | No | No | Yes | No | 2 | - |

*1 One score for each checkpoint:*

*Q1 Are both groups selected from the same and well-defined cohort?*

*Q2 Is the proportion of dropout in each group known, and if so, is it <15% in each?*

*Q3 Any comparison between full participants and those lost to follow-up?*

*Q4 Main potential confounders identified and considered?*

*Q5 Any confidence interval?*

*2.Grading was refined with a ‘+’ sign to suggest a low risk of bias for a score of 4 or 5, a ‘–’sign to suggest a high risk of bias for a score of 1 or 2, and no sign to suggest a moderate risk of bias for a score of 3.*

*Scottish Intercollegiate Guidelines Network. SIGN 50: a guideline developer’s handbook. Edinburgh, UK: SIGN, 2014*
